# From data to evidence: why administrative databases alone cannot measure real-world effectiveness in rare diseases – the case of chronic myeloid leukaemia

**DOI:** 10.1038/s41375-026-03037-7

**Published:** 2026-06-26

**Authors:** Sara Mucherino, Enrica Menditto, Valentina Orlando

**Affiliations:** 1 Department of Pharmacy, University of Naples Federico II, Naples, Italy; 2 CIRFF, Centre of Pharmacoeconomics and Drug Utilization Research, University of Naples Federico II, Naples, Italy

**Keywords:** Drug therapy, Translational research

In recent scientific and regulatory discourse, the terms *real-world data* (RWD) and *real-world evidence* (RWE) are often used interchangeably, yet this conflation is conceptually flawed and scientifically consequential. RWD are, by regulatory definition, data routinely collected during the healthcare delivery [1, 2]. Administrative databases represent one of their most pervasive and scalable forms. Hence, they cover entire populations, allow longitudinal follow-up, and are, in this sense, among the most population-representative real-world sources available. However, as Gale and Hochhaus addressed, the fact that such data are generated in routine practice does not automatically mean that they can support valid inference on the real-world effectiveness of treatment [1]. The translation of data into information, and of information into clinically meaningful knowledge, is not automatic. Failure to recognise this is one of the central methodological challenges in contemporary pharmacoepidemiology and drug utilization studies [3–5]. Health administrative databases are designed primarily for management and governance purposes: they support the reimbursement processes for medications and healthcare services, verify the appropriateness of prescriptions, and monitor the delivery of healthcare services. They were not designed, nor are they structured, to answer questions about therapeutic effectiveness. The abundance of population-level data does not bridge their structural absence of the clinical variables that confer meaning upon what is recorded. Methodologically, this creates a *semantic gap*: administrative data capture the “when” and the “how much”, but not the “why” or “with what clinical outcome”. This limitation is not a correctable defect; it is inscribed in the very nature of these systems and, for questions of therapeutic effectiveness, cannot be resolved without integration with clinical data, although rigorous drug utilization research remains essential to interpret what administrative data can reliably show [3, 4]. This limitation can sometimes be mitigated in conditions where therapeutic effectiveness may be reflected in administratively traceable outcomes, such as avoided hospitalisations, or preventable healthcare costs [6]. In chronic myeloid leukaemia (CML), however, this is far less feasible. CML is a rare myeloproliferative neoplasm in which treatment decisions and long-term disease control are guided by serial molecular monitoring rather than by outcomes that routinely generate administrative traces. In patients treated with BCR::ABL1 tyrosine kinase inhibitors (TKIs), clinically decisive response assessments include BCR::ABL1 transcript kinetics, achievement and maintenance of major and deep molecular responses, and eligibility for and outcome of treatment-free remission (TFR) [7, 8]. These are central to contemporary CML management, yet they do not map reliably onto hospitalisations, diagnostic codes, or other routine administrative markers [7, 8]. CML therefore represents a particularly informative case in which the gap between RWD and RWE becomes especially clear and empirically testable. This limitation becomes empirically visible when administrative data are interrogated using drug utilization methods rigorously.

Using an Italian prescription-based administrative database covering ~5.9 million inhabitants, corresponding to roughly 10% of the Italian national population, we reconstructed longitudinal treatment trajectories of BCR::ABL1 TKIs in an incident, population-based cohort of 609 patients with CML followed for up to six years (2017–2023). The database allowed reconstruction of initiation, switching, interruption, and restart of TKI therapy. Annual CML incidence ranged from 1.0 to 2.0 cases per 100,000 inhabitants, consistent with Italian national averages and European estimates [9, 10]. Within this cohort, 29.2% of patients switched TKI at least once during follow-up, and 25.3% experienced at least one treatment interruption. These are informative population-level measures of treatment pathways, but they are insufficient to establish their clinical meaning. The most revealing observation was that among those who interrupted TKI therapy, 90.3% subsequently restarted treatment after a mean interval of ~112 days. From administrative data alone, it is impossible to determine whether these events represent clinically managed treatment interruptions due to adverse events, planned TFR attempts that subsequently failed, successful TFR episodes requiring restart owing to molecular relapse, or unintentional non-adherence. Without linkage to clinical data, 90.3% is not an answer as it is not clinically interpretable.

Administrative data are well suited to population-level assessment of treatment use and healthcare utilisation. Anyway, Table 1 summarises what the administrative source could and could not capture in this CML cohort. The distinction does not reflect poor data quality or incomplete capture within the administrative database, but a structural limitation. The variables that define the clinical trajectory in CML, i.e., disease phase at diagnosis, prognostic scores, cytogenetic and molecular response, are absent by design [3–7]. The clinical consequences of this absence are not abstract. Consider three patients with identical administrative records: all start imatinib, all have a treatment gap of approximately four months, and all three subsequently restart imatinib. In the first, the gap reflects a temporary TKI interruption due to adverse events, without loss of MMR. In the second, it reflects a guideline-concordant TFR attempt in a patient with sustained deep molecular response; restart follows molecular relapse. In the third, it reflects poor adherence in a patient who had never achieved MMR; the restart follows loss of response detected on molecular monitoring. The same administrative trajectories thus maps onto clinically distinct scenarios, with different implications for governance, prescribing policy, and health technology assessment.

**Table 1:** Availability of key CML outcomes in administrative versus clinical databases.

Outcome	Clinical database	Administrative database
*Outcomes retrievable from both data sources*		
Age, sex	✓	✓
Comorbidities (e.g., Charlson index)	✓	✓
First-line TKI and treatment sequences	✓	✓
Switching between TKIs (rates and timing)	✓	✓
Treatment interruptions and gaps	✓	✓
Discontinuation (event and timing)	✓	✓
Overall survival/all-cause vital status	✓	✓
Healthcare resource use	**–**	✓
*Outcomes available in clinical databases only – not retrievable from administrative data*		
Disease phase at diagnosis (CML-CP vs progression in AD)	✓	✗
Prognostic scores (Sokal/ELTS)	✓	✗
Complete cytogenetic response (CCyR)	✓	✗
Major molecular response (MMR)	✓	✗
Deep molecular responses (MR4/MR4.5)	✓	✗
TFR eligibility criteria (per ELN recommendations)	✓	✗
TFR outcomes and MMR loss after treatment stop	✓	✗
Time to regain MMR after treatment restart	✓	✗
Progression to AD	✓	✗
CML-related mortality	✓	✗

*AD* advanced disease, *CCyR* complete cytogenetic response, *CML-CP* CML in chronic phase, *ELTS* EUTOS Long-Term Survival score, *MMR* major molecular response, *MR4/MR4.5* deep molecular responses (BCR::ABL1 ≤ 0.01% and ≤0.0032% on International Scale, respectively), *TFR* treatment-free remission, *TKI* tyrosine kinase inhibitor, ✓ available, ✗ not available, – partially or indirectly available.

In the incident cohort, male sex and dasatinib initiation were associated with earlier treatment interruption, whereas nilotinib initiation was associated with a lower interruption risk. These findings are broadly consistent with clinical registry data on dasatinib-associated toxicity and nilotinib tolerability in routine practice [11–13]. Yet even these signals remain clinically underdetermined without response data, toxicity data, and the reasons underlying treatment change.

Taken together, these findings show that administrative databases are indispensable for population-level outcomes research, but structurally insufficient for evaluating therapeutic effectiveness in rare diseases treated with molecularly targeted therapies [3, 4, 14]. The next necessary step is linkage infrastructure across complementary data sources. Pseudonymised record-linkage frameworks have already been described and implemented in oncology, enabling integration of administrative and clinical data [15, 16]. As illustrated in Fig. 1, this is precisely the architecture required in CML: prescription data provide the administrative backbone, whereas clinical databases supply the variables needed to interpret response, treatment change, long-term outcome, and TFR. Importantly, these clinical data are already generated in routine care through serial BCR::ABL1 monitoring, milestone-based response assessment, and eligibility evaluation for TFR according to ELN recommendations [7, 8]. What is still missing is not the data themselves, but the governance and infrastructure needed to connect them to administrative records in a standardised, reproducible, and privacy-compliant way. This gap is no longer methodologically negligible. Healthcare systems are generating unprecedented volumes of data on drug utilisation, prescribing patterns, and resource consumption. Regulatory agencies and policy makers rely increasingly on administrative data to assess prescribing appropriateness and to inform health technology assessment decisions [1, 3–5]. Governance decisions based on administrative data alone therefore risk being made without clinically decisive evidence. This creates a structural mismatch: governance operates at population scale, whereas the available data do not capture the clinical logic underlying individual treatment pathways [5, 14]. This mismatch is especially consequential in rare diseases as small populations limit statistical power, key therapeutic endpoints are often molecular, functional, or quality-of-life-based and therefore do not generate hospitalisations or administrative coding [17]. A patient with CML who achieves sustained deep molecular response and remains in TFR may never be hospitalised for leukaemia, generate no related hospitalisation codes, and consume no acute hospital resources, despite having achieved a highly favourable therapeutic outcome. Conversely, a patient who loses MMR after a TFR attempt and must restart therapy may be statistically indistinguishable, in administrative data, from one who simply interrupted treatment owing to non-adherence. This limitation is structural rather than technical: it does not depend on data accuracy, but on the structural absence of the variables that confer clinical meaning upon what is recorded [6, 7, 15]. Recognition of this specificity should be translated into concrete institutional commitment. We propose that CML, by virtue of its internationally standardised molecular monitoring framework, well-defined clinical endpoints, mature guideline infrastructure, and established collaborative networks such as GIMEMA and ELN [7, 8, 18], constitutes an ideal proof-of-concept setting for building this integrative infrastructure. Lessons learned in CML can then inform analogous frameworks in other rare haematological malignancies where the same tension between administrative tractability and clinical interpretability applies [19–23]. The question is no longer whether such integration is desirable, but whether the haematology community and its institutional partners are willing to build the infrastructure required to make it routine, standardised, and reproducible. A governance structure would be capable of monitoring the entire patient journey, from early diagnosis to long-term outcome management, translating data into actionable tools for health policy. We contend that this is among the most consequential methodological priorities in haematological oncology for the years ahead.

**Fig. 1:**
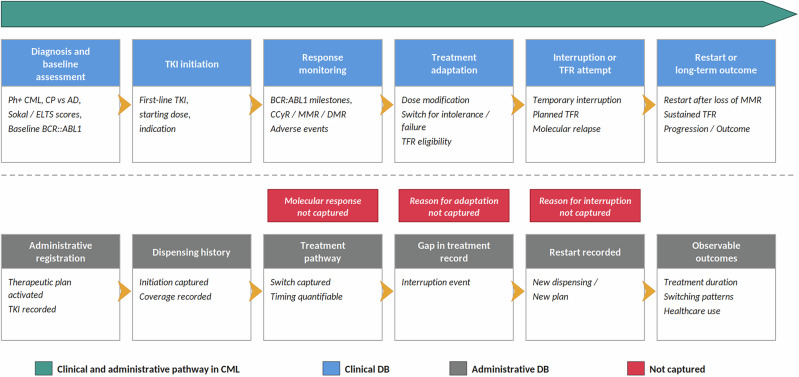
Clinical pathway and administrative trace in CML: where clinically decisive information is lost. AD advanced disease, BCR::ABL1 breakpoint cluster region-Abelson kinase fusion transcript, CCyR complete cytogenetic response, CML-CP chronic myeloid leukaemia in chronic phase, DMR deep molecular responses, ELTS EUTOS Long-Term Survival score, MMR major molecular response, Ph+ Philadelphia chromosome positive, TFR treatment-free remission, TKI tyrosine kinase inhibitor.

## Data Availability

The administrative data analysed in this study was based on prescriptions for medicines reimbursed by the National Health Service. The data source is based on therapeutical plans of the Campania Region, maintained by the Campania Regional Health Authority. Access was authorised to CIRFF under Regional Decrees DGRC n. 276 of 23/05/2017 and n.59 of 18/02/2026. Aggregated data are available from the corresponding author upon reasonable request, subject to data protection regulations and institutional data governance agreements.
